# *LmxM.22.0250*-Encoded Dual Specificity Protein/Lipid Phosphatase Impairs *Leishmania mexicana* Virulence *In Vitro*

**DOI:** 10.3390/pathogens8040241

**Published:** 2019-11-17

**Authors:** Natalya Kraeva, Tereza Leštinová, Aygul Ishemgulova, Karolina Majerová, Anzhelika Butenko, Slavica Vaselek, Julia Bespyatykh, Arzuv Charyyeva, Tatiana Spitzová, Alexei Yu. Kostygov, Julius Lukeš, Petr Volf, Jan Votýpka, Vyacheslav Yurchenko

**Affiliations:** 1 Life Sciences Research Centre, Faculty of Science, University of Ostrava, 71000 Ostrava, Czech Republic; luzikhina@gmail.com (N.K.); aishemgulova@gmail.com (A.I.); rolando24@yandex.ru (A.B.); arzuvc@gmail.com (A.C.); kostygov@gmail.com (A.Y.K.); 2 Department of Parasitology, Faculty of Science, Charles University, 12844 Prague, Czech Republic; Terka.Kratochvilova@seznam.cz (T.L.); k.majerova@email.cz (K.M.); slavica.vaselek@gmail.com (S.V.); tatiana.spitzova@gmail.com (T.S.); volf@cesnet.cz (P.V.); vapid@natur.cuni.cz (J.V.); 3 Biology Centre, Institute of Parasitology, Czech Academy of Sciences, 37005 České Budejovice (Budweis), Czech Republic; jula@paru.cas.cz; 4 Federal Research and Clinical Center of Physical-Chemical Medicine of Federal Medical Biological Agency, 119435 Moscow, Russia; juliabespyatykh@gmail.com; 5 Zoological Institute of the Russian Academy of Sciences, 199034 St. Petersburg, Russia; 6 University of South Bohemia, Faculty of Sciences, 37005 České Budejovice (Budweis), Czech Republic; 7 Martsinovsky Institute of Medical Parasitology, Tropical and Vector Borne Diseases, Sechenov University, 119435 Moscow, Russia

**Keywords:** LmDUSP1, virulence factor, *Leishmania* infection

## Abstract

Protein phosphorylation/dephosphorylation is an important regulatory mechanism that controls many key physiological processes. Numerous pathogens successfully use kinases and phosphatases to internalize, replicate, and survive, modifying the host′s phosphorylation profile or signal transduction pathways. Multiple phosphatases and kinases from diverse bacterial pathogens have been implicated in human infections before. In this work, we have identified and characterized the dual specificity protein/lipid phosphatase LmDUSP1 as a novel virulence factor governing *Leishmania mexicana* infection. The LmDUSP1-encoding gene (*LmxM.22.0250* in *L. mexicana*) has been acquired from bacteria via horizontal gene transfer. Importantly, its orthologues have been associated with virulence in several bacterial species, such as *Mycobacterium tuberculosis* and *Listeria monocytogenes*. *Leishmania mexicana* with ablated *LmxM.22.0250* demonstrated severely attenuated virulence in the experimental infection of primary mouse macrophages, suggesting that this gene facilitates *Leishmania* pathogenicity in vertebrates. Despite significant upregulation of *LmxM.22.0250* expression in metacyclic promastigotes, its ablation did not affect the ability of mutant cells to differentiate into virulent stages in insects. It remains to be further investigated which specific biochemical pathways involve LmDUSP1 and how this facilitates the parasite′s survival in the host. One of the interesting possibilities is that LmDUSP1 may target host′s substrate(s), thereby affecting its signal transduction pathways.

## 1. Introduction

The genus *Leishmania* (Kinetoplastea, Trypanosomatidae) contains parasitic flagellates causing leishmaniasis. This disease is clinically pleomorphic, varying from fairly harmless self-healing skin lesions to fatal visceral organ failure [1]. *Leishmania* spp. are dixenous parasites, i.e. they have two different hosts in their life cycle [2,3]. The flagellated extracellular promastigotes develop in the gut of insect vectors, while the intracellular non-flagellated amastigotes occupy the phagolysosomes of vertebrates′ macrophages [4,5]. Genes and gene products, governing differentiation or infection maintenance, are putative virulence factors. At present, they are identified primarily by the next-generation sequencing (NGS)-based approaches [3,6] and analyzed using functional genomics [7,8,9,10,11]. 

One of the genes previously identified in our analyses is *LmxM.22.0250*. Its orthologues were upregulated in metacyclic promastigotes of *Leishmania mexicana* M379 and in a virulent strain (as compared to its avirulent kin) of *Leishmania major* LV561 [12]. Confirming this and highlighting its potential importance, *LmxM.22.0250* orthologue was one of only four genes consistently upregulated in both fly-derived and axenically differentiated metacyclic promastigotes of *L. major* [13]. This gene is present in most trypanosomatid lineages, genomes of which are available from the TriTrypDB [14]. Remarkably, it appears to be selectively lost from the genomes of *Trypanosoma* and *Blechomonas* spp. [15], while its distribution within Leishmaniinae is not uniform. Orthologues of *LmxM.22.0250* are pseudogenized in some *Leishmania* species or strains [16].

*LmxM.22.0250* encodes a dual specificity phosphatase (DUSP), which we named LmDUSP1. In general, such enzymes can recognize and work upon more than one molecular substrate [17]. Specifically, LmDUSP1 can dephosphorylate both proteins and lipids [16,18]. It contains a conserved P-loop signature sequence HCXXGKDR, governing substrate binding and its subsequent dephosphorylation [19]. This enzyme can remove phosphates from phospho-tyrosine (P-Tyr) and phospho-serine (P-Ser) at the rates of 12 and 0.6 nmoles/mg min, respectively, indicating that it has a higher preference towards P-Tyr. In addition, it can also dephosphorylate different mono-phosphorylated inositols at the rate of about 10 nmoles/mg min [18]. Of note, all these biochemical activities have been measured in vitro, and it remains to be established what role LmDUSP1 plays under physiological conditions. Another lipid phosphatase (encoded by *LbrM.25.21.80*) may contribute to the parasite virulence, as it was shown to be among the 100 most abundantly expressed genes in *Leishmania braziliensis* skin lesions of humans with cutaneous leishmaniasis [20].

It has been previously shown that LmDUSP1 has bacterial origin and is similar to several proteins identified as virulence factors in some pathogenic microorganisms [16,18]. The most notable examples are secreted phosphatases MptpB from *Mycobacterium tuberculosis* [21] and LipA from *Listeria monocytogenes* [22]. Two secreted tyrosine phosphatases of *M. tuberculosis*, MptpA and MptpB, play an important role in the interaction of the pathogen with the host cell [23]. While MptpA homologs are present in both pathogenic (*M. tuberculosis* complex) and non-pathogenic (e.g. *Mycobacterium smegmatis*) mycobacteria, MptpB is restricted to members of the former complex. Importantly, mycobacteria with inhibited MptpB or ablated *mptpB* gene are severely defective in their ability to survive in activated mouse macrophages and in guinea pigs [24,25]. It has been hypothesized that MptpB dephosphorylates the P-Tyr residues of the myelin basic protein, thus enabling the mycobacteria to survive within its host [23,26]. Similarly, the virulence of *Listeria monocytogenes* strains lacking the *lmo1800* gene, which encodes a secreted tyrosine phosphatase LipA, is severely attenuated in vivo [22].

In this work, we investigated the role of LmDUSP1 in *Leishmania* virulence both *in vitro* and *in vivo*. 

## 2. Results

### 2.1. *LmxM.22.0250* Encodes a Dual Specificity Phosphatase LmDUSP1: *in Silico* Analyses

**Figure 1:**
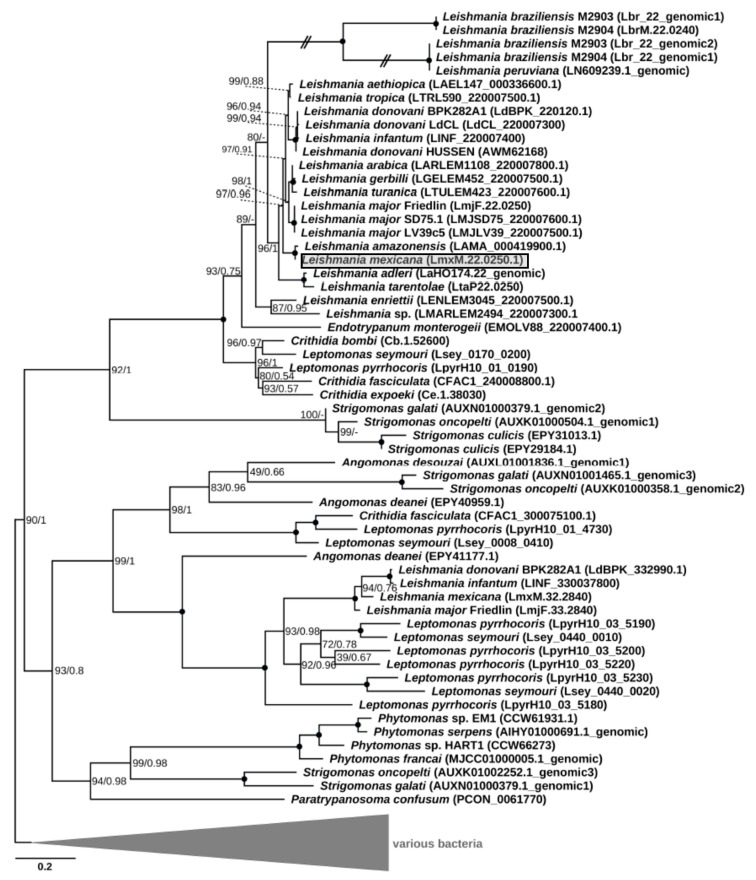
Maximum-likelihood phylogenetic tree of *LmxM.22.0250* and its homologs. Numbers at the nodes represent bootstrap percentage/posterior probability. Nodes with maximal support are marked with black circles. Dashes indicate different topology in the Bayesian tree. The scale bar shows the number of substitutions per site. Double-crossed branches are shown at 25% of their actual length. The gene of interest is boxed and shaded. The tree is rooted with bacterial sequences. In cases where a sequence was not annotated in the genome and could be identified only using TBLASTN, a sequence ID is in the following format: chromosome#_genomic.

Phylogenetic analysis revealed clustering of the putative *LmxM.22.0250* homologs into four clades. One of them included the gene under study along with the sequences identified in all other Leishmaniinae, whose genomes had been sequenced thus far, and those of the genus *Strigomonas* from the endosymbiont-harboring subfamily Strigomonadinae (Figure 1). Of note, all recognized subgenera of *Leishmania* (*Leishmania*, *Viannia*¸ *Sauroleishmania*, and *Mundinia* [27,28]) were included in the analysis, but the homologous sequences of the *L.* (*Viannia*) species were pseudogenized. The three other clades composed a super-clade, which was sister to the first one. Only one of these three clades contained sequences of *Leishmania* spp., which were exclusively from the members of the nominative subgenus *L.* (*Leishmania*). In addition, this clade included one gene from *Leptomonas pyrrhocoris* and one from a single member of Strigomonadinae, *Angomonas deanei.* The third and fourth clades contained genes of Strigomonadinae and monoxenous Leishmaniinae, and those of *Strigomonas*, *Phytomonas* spp., and *Paratrypanosoma confusum*, respectively. The inferred phylogeny of DUSPs and, in particular, the absence of this gene in the free-living eubodonid *Bodo saltans* and its presence in various clades, including the earliest branching *P. confusum*, suggests that the acquisition this gene occurred via lateral gene transfer from a bacterium in the last common ancestor of all trypanosomatids. Subsequently, it experienced at least three ancient duplications, which occurred not later than in the common ancestor of Leishmaniinae and Strigomonadinae, and multiple independent losses in different lineages of Trypanosomatidae. In trypanosomes and monoxenous parasites of fleas (*Blechomonas* spp.), these losses resulted in a complete ablation of the DUSP-encoding genes.

It is not clear whether the genes falling into different clades have the same function, since the corresponding proteins are characterized by rather low sequence identity, even within the same species. For example, the amino acid sequence identity between the two paralogs found in *L. mexicana* (*LmxM.22.0250* and *LmxM.32.2840*) is just ~28%. In all the analyzed sequences except for the pseudogenes of *L.* (*Viannia*), a tyrosine phosphatase domain was clearly identifiable.

### 2.2. Conventional Genetic Ablation of *LmxM.22.0250*

To examine whether LmDUSP1 is involved in *L. mexicana* virulence, we first subsequently deleted two alleles of *LmxM.22.0250* by replacing them with antibiotic resistance genes for Sat and Hyg (Figure S1A). Similarly to the case of LmxBTN1 reported previously [9], Southern blotting showed that *LmxM.22.0250* ablation was not complete (Figure S1B, panel 5′ UTR). We concluded that at least one extra copy of this gene must be present elsewhere in the *L. mexicana* genome. Whether this is a naturally occurring phenomenon or a result of genetic manipulations remains to be examined further. 

### 2.3. CRISPR-Cas9-mediated Genetic Ablation of *LmxM.22.0250*

**Figure 2:**
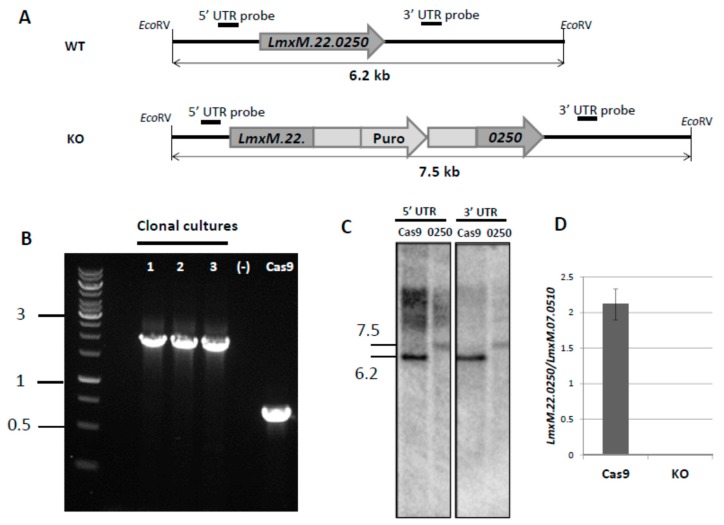
Knock-out of *LmxM.22.0250* with CRISPR-Cas9 approach. (**A**) Schematic depictions of the WT (Cas9) and recombined alleles after replacement with puromycin-resistant gene; annealing positions of the probes and expected fragment sizes are shown. (**B**) PCR analysis of LmDUSP1 KO clonal cultures, a wild type (Cas9), and a negative control; 1 kb DNA ladder is on the left. (**C**) Southern blotting results of the *Eco*RV digested *L. mexicana* genomic DNA of the wild type (Cas9, labelled Cas9) and *LmxM.22.0250* ablated strains (clonal culture 1 from 2B, labelled 0250) with *LmxM.22.0250* 5’ UTR and *LmxM.22.0250* 3’ UTR probes. (**D**) Quantitative RT-PCR analysis of *LmxM.22.0250* gene expression in the wild type (Cas9) and LmDUSP1 KO *L. mexicana*. Gene expression was normalized to *LmxM.07.0510* [29] and presented as means and standard deviations of three independent biological replicates. Sizes in (**B**,**C**) are in kb.

In order to avoid the hurdles with *LmxM.22.0250* conventional knock-out, we decided to use the CRISPR-Cas9 system, previously established in our lab [9], to ablate this gene in *L. mexicana* (Figure 2). The gRNA under control of the U6 promoter in forward orientation was stably expressed from the *L. mexicana* 18S ribosomal DNA locus. PCR (Figure 2B), Southern blotting (Figure 2C), and qRT-PCR (quantitative Reverse Transcription Polymerase Chain Reaction) data (Figure 2D) confirmed complete ablation of the *LmxM.22.0250* gene. The resulting strain was named LmDUSP1 KO. 

### 2.4. Growth Kinetics of *Leishmania* Strains *In Vitro*

**Figure 3:**
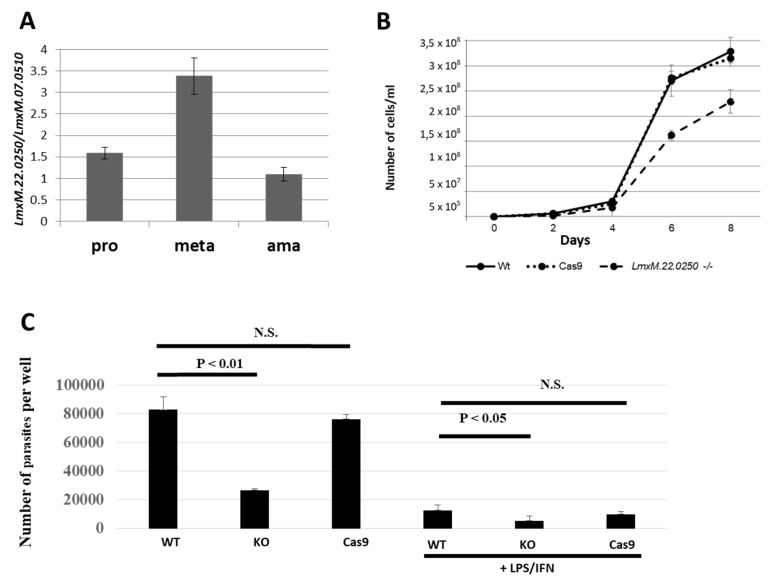
Expression of *LmxM.22.0250* in *L. mexicana* development, growth kinetics, and macrophage infection in vitro of the wild type (WT), Cas9, and LmDUSP1 KO cells. (**A**) Quantitative RT-PCR analysis of *LmxM.22.0250* expression in the axenically differentiated procyclic promastigotes (pro), metacyclic promastigotes (meta), and amastigotes (ama) of the wild type (Cas9) *L. mexicana*. (**B**) Growth curves of the WT, Cas9, and LmDUSP1 KO *L. mexicana*. (**C**) The intensity of infection (number of parasites per well) was calculated for WT, LmDUSP1 KO, and Cas9-expressing *L. mexicana* in non-stimulated or LPS/IFN-γ stimulated primary murine macrophages. Data are summarized from three independent biological replicates. The error bars indicate standard deviation. N.S. indicates a not statistically significant difference.

We first investigated the expression profile of *LmxM.22.0250* in the wild type *L. mexicana*. In agreement with previous reports [12,13], we documented a significant upregulation of its expression in axenically differentiated metacyclic promastigotes (Figure 3A). We propose using this gene as a marker of metacyclogenesis in *Leishmania* (*Leishmania*) spp. 

Next, we studied the effect of *LmxM.22.0250* ablation on *L. mexicana* growth by comparing cell division of the wild type, Cas9, and LmDUSP1 KO strains in vitro. To eliminate the negative effect of continuous in vitro cultivation [30], the procyclic promastigote cultures were started from the cells passaged through animals. Growth kinetics were monitored every 48 hours. Our data demonstrated that the promastigotes of the LmDUSP1 KO strain divide significantly slower compared to their wild type or Cas9 counterparts (Figure 3B). This was reminiscent of the situation previously documented for *L. mexicana* with ablated *LmxM.30.2090*, a gene encoding a putative ATP/GTPase ALD1 [31]. 

In addition, we also checked whether the absence of *LmxM.22.0250* can be compensated by overexpression of its paralog, *LmxM.32.2840*. The expression level of *LmxM.32.2840* was assayed by qRT-PCR in *Leishmania* developmental stages (Figure S2). No statistically significant difference was detected between the wild type (Cas9) and LmDUSP1 KO cells, suggesting that *LmxM.32.2840* does not compensate for the loss of *LmxM.22.0250* function.

### 2.5. Experimental Infection of *Lutzomyia longipalpis*

In total, 215 *Lutzomyia longipalpis* females were dissected, out of which 68 were infected with the wild type, 76 with Cas9, and 71 with LmDUSP1 KO *L. mexicana*. On day 2 PBM (post blood meal), infection rates and intensity were similar between the tested lines (p = 0.85 and p = 0.92, respectively; Figure S3A). In all lines, procyclic promastigotes were localized inside the endoperitrophic space, within the bloodmeal surrounded by the peritrophic matrix (Figure S3B). On day 8 PBM, fully developed late infections were documented in all three lines, with similar infection rates ranging from 84% to 91% (p = 0.56) (Figure S3A). Heavy infections (62%) dominated in females harbouring the wild type strain, while moderate infections were predominant in females infected with Cas9 (53%) and LmDUSP1 KO (48%) *Leishmania* strains. No differences were documented in the localization of infections on day 8. Parasites of all tested strains were always present in the abdominal and/or thoracic midgut. Colonization of the stomodeal valve was observed in a similar percentage in all three strains: 83%, 82%, and 86% for the wild type, Cas9, and LmDUSP1 KO, respectively (Figure S3B).

### 2.6. Macrophage Infection *In Vitro*

We also investigated whether LmDUSP1 KO can manifest virulence defects in cultured primary murine macrophages. Non-activated and classically stimulated macrophages were compared side-by-side in infection experiments using wild type, Cas9 control, and LmDUSP1 KO strains of *L. mexicana*. In both cases, the levels of the LmDUSP1 KO infections were significantly lower compared to those of wild type or Cas9 flagellates (Figure 3C). Thus, we concluded that wild type and LmDUSP1 KO strains differ in their ability to infect or develop in the primary murine macrophages in vitro.

### 2.7. Infection of Mice with LmDUSP1 KO L. mexicana

**Figure 4:**
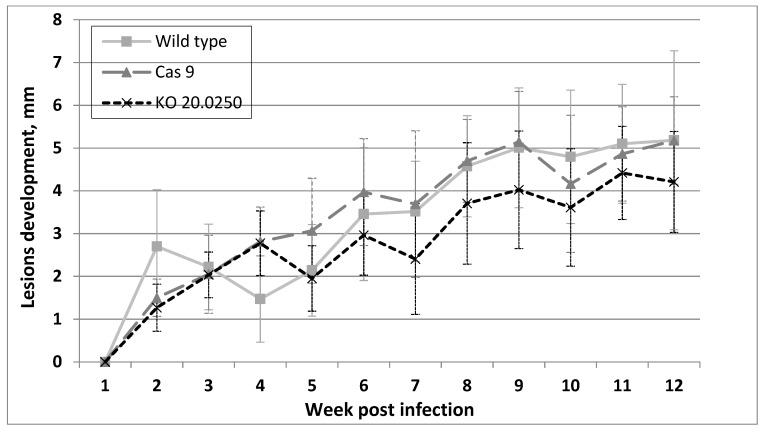
Development of clinical symptoms in inoculated mice ears. Diameter of the lesions from seven independent experiments per *Leishmania* strain, measured weekly. Data for one representative experiment (out of two) are presented.

All BALB/c mice inoculated with the wild type, Cas9, and LmDUSP1 KO strains developed lesions. In the LmDUSP1 KO strain, lesions were somewhat smaller than in the wild type or Cas9 controls starting from week 7 post infection, although the variation between individual animals (14 per strain, 42 in total) did not allow us to assign statistical significance to this observation (Figure 4). 

The effect of *LmxM.22.02250* ablation remains to be investigated in other animal models (for example, hamster, dog, non-human primate, or wild rodent [32]) available to researchers in the field. 

## 3. Discussion

Protein phosphorylation/dephosphorylation is a vital regulatory mechanism controlling cellular metabolism, growth, trafficking, and other important physiological processes. The sets of cellular kinases and phosphatases perform reversible phosphorylation of proteins on Ser, Thr, and Tyr. Protein Tyr phosphatases are a large superfamily that encompasses the most divergent dual specificity phosphatases [33]. Numerous pathogens successfully use kinases and phosphatases to internalize, replicate, and survive, modifying the host′s phosphorylation profile or signal transduction pathways [34]. Multiple phosphatases and kinases from diverse bacterial pathogens such as *Salmonella*, *Yersinia*, *Listeria*, and *Mycobacterium* spp. have been previously identified as virulence factors in human infections [22,35,36,37]. 

Recently, protein tyrosine phosphatases have been implicated in *Leishmania* pathogenesis [38,39]. In this work, we identified and characterized a novel putative virulence factor of *Leishmania* infection. This dual specificity protein/lipid phosphatase (LmDUSP1) can dephosphorylate two types of molecules; in proteins, it recognizes preferentially P-Tyr, but also P-Ser. The LmDUSP1-encoding gene (*LmxM.22.0250* in *L. mexicana*) was acquired from bacteria via horizontal gene transfer. Importantly, its orthologues have been implicated in virulence in several bacterial species, e.g. *M. tuberculosis* [21] and *L. monocytogenes* [22]. 

In trypanosomatids, these genes were completely lost from some basal lineages (most notably, *Trypanosoma* and *Blechomonas*), while retained in others (monoxenous and dixenous Leishmaniinae, *Phytomonas* spp., Strigomonadinae, and early-branching *Paratrypanosoma*) [2]. Moreover, Strigomonadinae and Leishmaniinae harbor multiple anciently-diverged paralogues in the genome, suggesting a significant separation of DUSPs′ functions. Our data, demonstrating that ablation of *LmxM.22.0250* does not change the expression of the paralogous *LmxM.32.2840* gene, are in agreement with this hypothesis. We also noted that the DUSP1-encoding gene has been pseudogenized in members of the subgenus *Leishmania* (*Viannia*), suggesting that these parasites rely on other factors determining their virulence [40,41]. 

*Leishmania mexicana* with ablated *LmxM.22.0250* demonstrated attenuated virulence in mouse macrophages, suggesting that this gene is important for infectivity in vertebrates. Despite significant upregulation of *LmxM.22.0250* expression in metacyclic promastigotes (Figure 3A), its ablation did not affect the ability of mutant cells to differentiate into the virulent stages. In contrast to the "ancestral" bacterial secreted phosphatases, no signal peptide sequence was found in LmDUSP1. This is reminiscent of the situation of another *Leishmania* protein tyrosine phosphatase implicated in virulence, LPTP1. This protein also does not encode a signal peptide and is not secreted [39]. Another possibility is that LmDUSP1-encoded phosphatase is secreted by means of the non-classical secretion pathways, which might play a major role in yet understudied leishmanial protein secretion [42].

It remains to be investigated which specific biochemical pathways involve LmDUSP1 and how this facilitates parasites survival in the mammalian host. One of the interesting possibilities is that LmDUSP1 may target substrate(s) of the host, thereby affecting its signal transduction pathways.

## 4. Materials and Methods 

### 4.1. *In Silico* Analyses 

For the identification of the *LmxM.22.0250* homologs, BLASTP and TBLASTN searches were performed using this protein as a query for a local database containing all trypanosomatid annotated proteins and genome assemblies available in the TriTrypDB (release 35) [14] and NCBI Genomes, as well as the genome of the eubodonid *Bodo saltans* (Table S1). The closest homologs outside Kinetoplastea were identified using a BLASTP search against NCBI nr database. Duplicates and sequences from organisms without clear taxonomic identification were removed from the best 500 collected hits. The remaining 139 protein sequences were combined with the trypanosomatid hits and aligned using the L-INS-i algorithm in MAFFT v. 7.4 [43]. Poorly aligned regions were removed using trimAl v. 1.4 [44] with the default settings, resulting in the final alignment containing 189 positions. The maximum likelihood phylogenetic tree was inferred in IQ-TREE v. 1.68 [45] with the LG + I + G4 model, as selected by the built-in ModelFinder module [46], and 1,000 standard bootstrap replicates. Bayesian inference was conducted using MrBayes v. 3.2.6 [47] with analysis run for 1 million generations, sampling every 100th of them. The model of the site heterogeneity (I + G) was set based on the ModelFinder analysis, while the optimal model of the amino acid substitutions (Wag) was selected in MrBayes using the mixed amino acid model prior. Domain prediction was carried out using a search against the Pfam database [48].

### 4.2. Axenic Cultivation and Growth Kinetics

*Leishmania mexicana* (isolate MNYC/BZ/62/M379) culture was maintained in M199 medium (Sigma-Aldrich, St. Louis, USA) supplemented with 2 μg/mL Biopterin (Sigma-Aldrich), 2 μg/mL Hemin (Jena Bioscience GmbH, Jena, Germany), 25mM HEPES, 50 units/mL of penicillin, 50 μg/mL of streptomycin, and 10% fetal bovine serum (FBS) (all from Life Technologies, Carlsbad, USA) at 23 °C. Analysis of growth kinetics was done as described previously [9]. All cell lines were passaged through mice before further analyses. For this, one mouse per each strain was intradermaly infected by injection into the base of tail. At week 9 post infection, all three *Leishmania mexicana* strains (wild type, LmDUSP1 KO, and Cas 9) were re-isolated into the culture. Expression of *LmxM.22.0250* and *LmxM.32.2840* was analyzed by RT-qPCR using primer pairs LmxM.22.0250_qPCR_f2 and LmxM.22.0250_qPCR_r2, and LmxM.32.2840_qPCR_f and LmxM.32.2840_qPCR_r, respectively (Table S2) and normalized as in [29]. 

### 4.3. Genetic Manipulations of *Leishmania mexicana*: Conventional Approach 

In order to ablate *LmxM.22.0250* in *L. mexicana*, two alleles were sequentially replaced with selectable markers for Nourseothricin (Sat) and Hygromycin (Hyg). Targeting constructs were generated by fusion PCR [49]. In the first round of PCR, 5′ and 3′ arms of homology were amplified from the *L. mexicana* genomic DNA using primers A/B and D/F, respectively (Table S2). The ORFs of the Sat- and Hyg-resistance genes were amplified from the plasmids pF4T7polNLS1.4sat and pF4TR1.4hyg [50], using primer pairs SAT_5′f/SAT_3′r and Hyg_5′f/Hyg_3′r, respectively [9]. In the fusion PCR reaction, 5′ and 3′ arms of homology were combined with either the Sat- or Hyg-resistance gene and amplified with nested primers G and H (Table S2). *L. mexicana* promastigotes were transfected with 5 μg of the targeting constructs as described previously using a BTX ECM 630 electroporator (Harvard Apparatus Inc, Holliston, USA) [51]. The first allele knockout cell line was isolated in complete M199 medium containing 100 μg/mL of Sat (Jena Bioscience). The second allele knockout *L. mexicana* clones were selected on solid M199 medium supplemented as above with additional 100 μg/mL of Sat and 100 μg/mL of Hyg. Correct integration was confirmed by Southern blot [52]. In brief, total genomic DNA was isolated using a DNeasy Blood & Tissue Kit (Qiagen, Hilden, Germany), digested with *Eco*RV overnight, separated on 0.75% agarose gel, and transferred to a ZetaProbe blotting membrane (Bio-Rad, Hercules, USA). The following primers (Table S2) were used to amplify probes: SBp_SAT_f and SBp_SAT_r (for Sat), SBp_Hyg_f and SBp_Hyg_r (for Hyg), South5_LmxM.22.0250_f and South5_LmxM.22.0250_r (for 5′ UTR). The probes were labelled with radioactive ^32^P using the DecaLabel DNA Labeling kit (Thermo Fisher Scientific, Waltham, USA).

### 4.4. Genetic Manipulations in *Leishmania mexicana*: CRISPR-Cas9 

In order to ablate *LmxM.22.0250,* we used a strategy described earlier [9] with gRNA gtgatgaagcaggtatcgat|cgg. The U6 promoter, gRNA with tracrRNA, and U6 terminator were amplified using the following primers, respectively (Table S2): A_U6_prom_LmxM_f and B_LmxM.22.0250-sgRNA281_r, C_U6_term_LmxM_f and D_U6_term_LmxM_r, E_LmxM.22.0250-sgRNA281_f and F_sgRNA_mCh396_r. These fragments were fused with primers G_sgRNA_NotI_f and H_sgRNA_NcoI_r, and cloned into pLEXSY-SAT2 (Jena Bioscience). The donor sequence encoding the puromycin (Puro) resistance gene was amplified from pLS6-PFR2 [10] with 30 bp of LmxM.22.0250 sequences flanking double-stranded break site using primers I_donor_LmxM.22.0250-281_f and J_donor_LmxM.22.0250-281_r. The construct containing gRNA and the donor construct were transfected into the CRISR-Cas9-expressing *L. mexicana* [9]. Clones were selected on solid complete M199 medium supplemented with 100 μg/mL of Hyg, 100 μg/mL of Sat, and 20 μg/mL of Puro. Ablation of *LmxM.22.0250* was verified by PCR with primers LmxM.22.0250_check_1f and LmxM.22.0250_check_1r (wild type size is 792 bp; after donor insertion the size is 2,045 bp), and by Southern blot as described earlier with probes 5′ UTR-, 3′ UTR-, and Puro. The following primers were used for probes amplification: Puromycin probe: South_Puro_f and South_Puro_r; 5’UTR probe: South5_LmxM.22.0250_f and South5_LmxM.22.0250_r, 3’UTR probe: South6_LmxM.22.0250_f and South6_LmxM.22.0250_r (Table S2). 

### 4.5. Infection of Macrophages

Parasites were harvested in the stationary phase, washed three times in the 0.9% saline solution (5 min, 6,000 rpm), re-suspended in the complete macrophage´s medium (see below), and their concentration was determined by hemocytometer.

Bone marrow was obtained by flushing the tibiae and femurs of euthanized BALB/c mice. The differentiation from bone marrow precursor cells to bone marrow-derived macrophages proceeded for 7 days at 37 °C with 5% CO_2_ in the presence of the L929 fibroblast cell culture supernatant (20%), which served as a source of the macrophage colony-stimulating factor. After differentiation, the macrophages were cultured as above in the complete RPMI-1640 medium supplemented with 10% FBS, 50 units/mL penicillin, 50 μg/mL streptomycin, 2 mM L-glutamine, and 0.05 mM 2-mercapto-ethanol (all from Sigma-Aldrich). 

The bone marrow-derived macrophages were plated into CellStar 24-wells (Greiner Bio-One GmbH, Kremsmünster, Austria) at a concentration of 4 × 10^5^ cells/ml. The stationary-phase *Leishmania* cells were added at a parasite-to-macrophage ratio of 6:1. After two hours, the cells were washed and incubated either in the complete RPMI 1640 or in the media combined with 50 U/mL IFN-γ (Bio-Rad) and 0.5 µg/mL LPS (Sigma-Aldrich) (classically stimulated macrophages). At 72 hours post infection, macrophages were lysed in 0.016% SDS (Sigma-Aldrich) and washed with 0.9% saline solution, and amastigotes were spun down by centrifugation, re-suspended in the complete RPMI medium, and counted with a hemocytometer. All experiments were performed in two independent biological replicates, and samples were analyzed in triplicate.

### 4.6. Experimental Infection of *Lutzomyia longipalpis*

For all the experiments, a well-established sand fly colony of *Lutzomyia longipalpis* (originated from Brazil) was used. This colony was maintained under standard conditions as described previously [53,54]. At 24 hours prior to the infection feeding, groups of approximately 150 sand fly females (5–7 days old) were separated and deprived of sucrose food. Promastigotes from log-phase cultures were resuspended in heat-inactivated sheep blood at concentration of 10^6^ cells/mL and offered to sand flies through the chicken skin. Engorged females were separated, provided with sucrose, and maintained under standard conditions until the end of the experiment. 

Dissections were performed before defecation (early stage of infection) on day 2 post blood meal (PBM) and after defecation (late stage of infection) on day 8 PBM. Abundance and localization of the flagellates in the sand fly gut were examined by light microscopy. Parasite loads were graded as light, moderate/medium, and heavy (≤100, 100–1000, and ≥1000 parasites per gut, respectively) as described previously [55]. Experimental feeding of *L. longipalpis* was repeated three times for each *L. mexicana* line. 

### 4.7. Mice Infection

In order to investigate the ability of parasites to infect the mammalian host, BALB/c mice were experimentally infected with the freshly isolated wild type, LmDUSP1 knock-out (KO), and Cas 9 *L. mexicana* cells. Suspensions of 5 × 10^5^ parasites in 5 µl of saline solution were injected intradermally into the ear pinnae of mice anesthetized by ketamine/xylazin (62.5 mg/kg and 25 mg/kg, respectively). The experiments were repeated twice, and seven mice for each *Leishmania* strain were used every time. Development of clinical symptoms and size of nodular lesions were measured weekly. Mice were sacrificed at the 14th week post infection in the first experiment and 12th week post infection in the second experiment. Their infected ears were dissected, and parasites were re-isolated into the culture. 

Ethics statement: Animals were maintained and handled in the animal facility of Charles University in Prague in accordance with institutional guidelines and Czech legislation (Act No. 246/1992 and 359/2012 coll. on protection of animals against cruelty in present statutes at large), which complies with all relevant EU guidelines. All the experiments were approved by the Committee on the Ethics of Laboratory Experiments of the Charles University and were performed under permission No. MSMT-31114/2015-13 of the Czech Ministry of the Environment. All efforts were made to minimize the number and suffering of experimental animals during the study.

### 4.8. Statistical Analysis

Statistical analysis was carried out using R software (http://cran.r-project.org). Infection rates and intensity of infection were analysed using Fisher´s exact test and proportion test, respectively. A p-value of <0.05 was considered statistically significant.

## Supplementary Materials

The following are available online at https://www.mdpi.com/2076-0817/8/4/241/s1, **Figure S1**: Conventional ablation of *LmxM.22.0250*. A: Schematic representation of the WT and recombined alleles after replacement with Sat- and Hyg-resistant genes; annealing positions of the probes and expected fragment sizes are shown. B: Southern blot analysis of the *Eco*RV digested *L. mexicana* genomic DNA of the wild type (WT) and *LmxM.22.0250* ablated strains (clonal cultures 1 and 2) with Sat, Hyg, and *LmxM.22.0250* 5’ UTR probes. Indicated sizes are in kb. **Figure S2**: Expression of *LmxM.32.2840* in *L. mexicana* developmental stages. **Figure S3**: Experimental infection of *Lutzomyia longipalpis*. A: Infection rates and intensity in *L. longipalpis* harbouring different strains of *L. mexicana*; B: Localization of parasites inside *L. longipalpis* gut after infection with different strains of *L. mexicana*. **Table S1**: Kinetoplastid genome sequences analyzed in this study. **Table S2**: List of primers used in this study for amplification of the *LmxM.22.0250* gene specific targeting sequences, selectable markers, fusion PCR products, Southern blot probes, and PCR analysis of correct integration.
