# Supplementary material for: LmxM.22.0250-Encoded Dual Specificity Protein/Lipid Phosphatase Impairs Leishmania mexicana Virulence In Vitro
Source: Pathogens. 2019 Nov 17;8(4):241. doi: 10.3390/pathogens8040241 (PMC6969907; doi:10.3390/pathogens8040241)
Supplement: Supplementary file 1 [file pathogens-08-00241-s001.zip › Suppl Fig 1.pptx]

## Slide 1
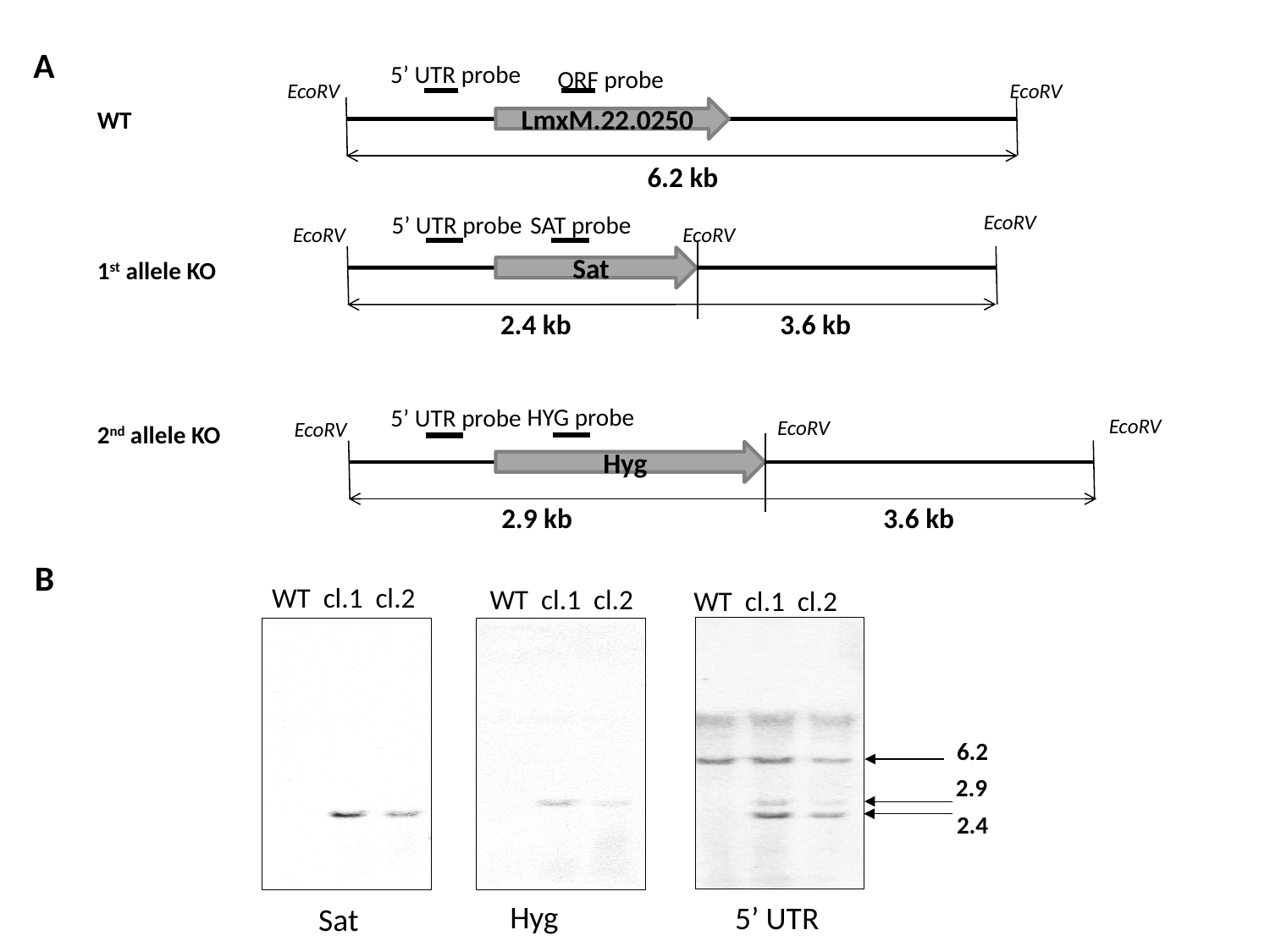

A
5’ UTR probe
ORF probe
EcoRV
EcoRV
LmxM.22.0250
6.2 kb
WT
EcoRV
SAT probe
Sat
2.4 kb
EcoRV
EcoRV
1st allele KO
3.6 kb
EcoRV
EcoRV
EcoRV
2nd allele KO
Hyg
2.9 kb
3.6 kb
5’ UTR probe
HYG probe
5’ UTR probe
B
WT cl.1 cl.2
WT cl.1 cl.2
WT cl.1 cl.2
6.2
2.9
2.4
Hyg
5’ UTR
Sat
