# Supplementary figures and images for: LmxM.22.0250-Encoded Dual Specificity Protein/Lipid Phosphatase Impairs Leishmania mexicana Virulence In Vitro
Source: Pathogens. 2019 Nov 17;8(4):241. doi: 10.3390/pathogens8040241 (PMC6969907; doi:10.3390/pathogens8040241)

## Slide 1
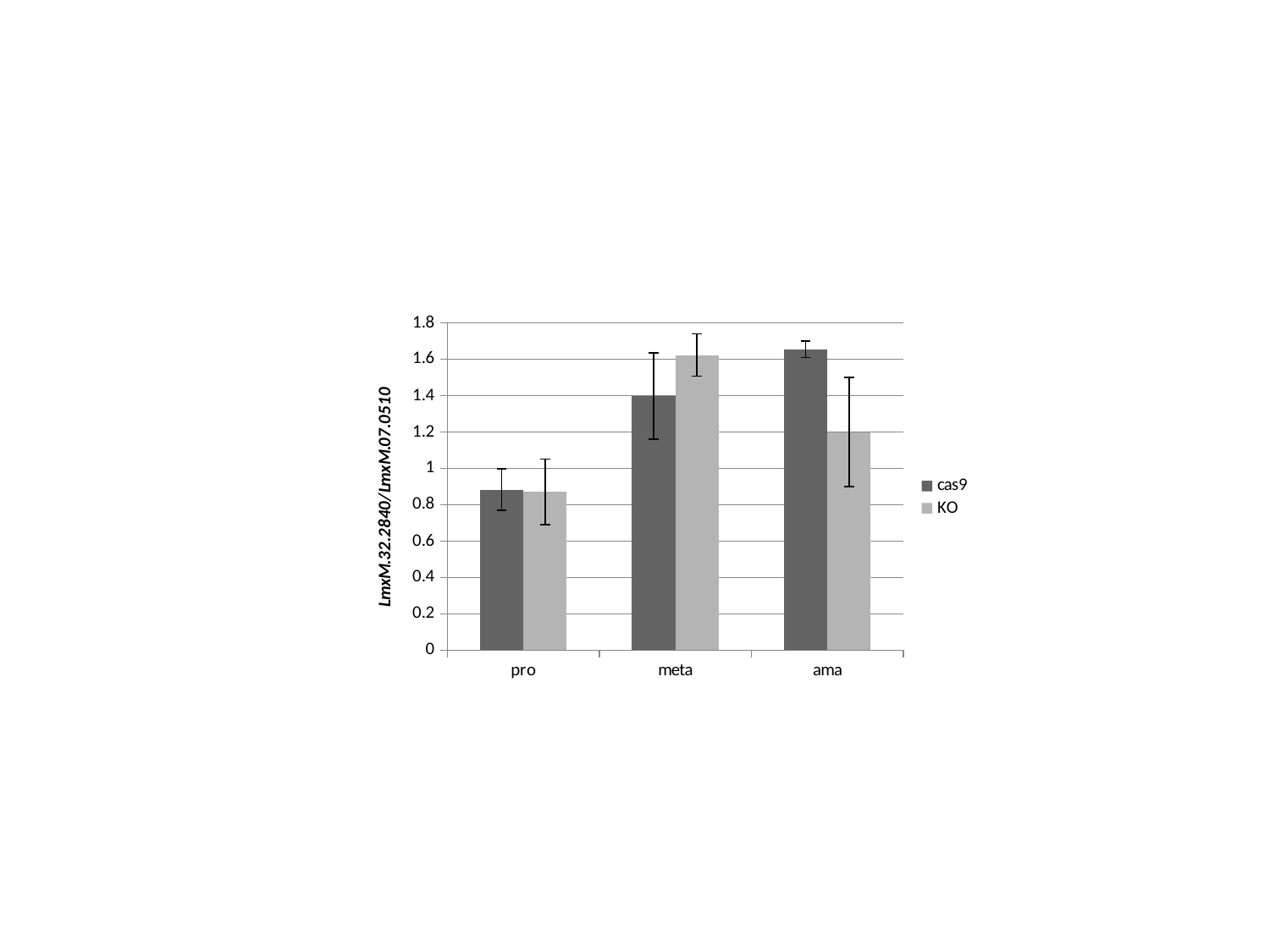

### Chart
| Category | cas9 | KO |
|---|---|---|
| pro | 0.8835477067057639 | 0.8708067449658753 |
| meta | 1.3988653015728119 | 1.623728819178991 |
| ama | 1.6562911950948915 | 1.199320729610643 |

Supplement: Supplementary file 1 [file pathogens-08-00241-s001.zip › Suppl Fig 2.pptx]

## Slide 1
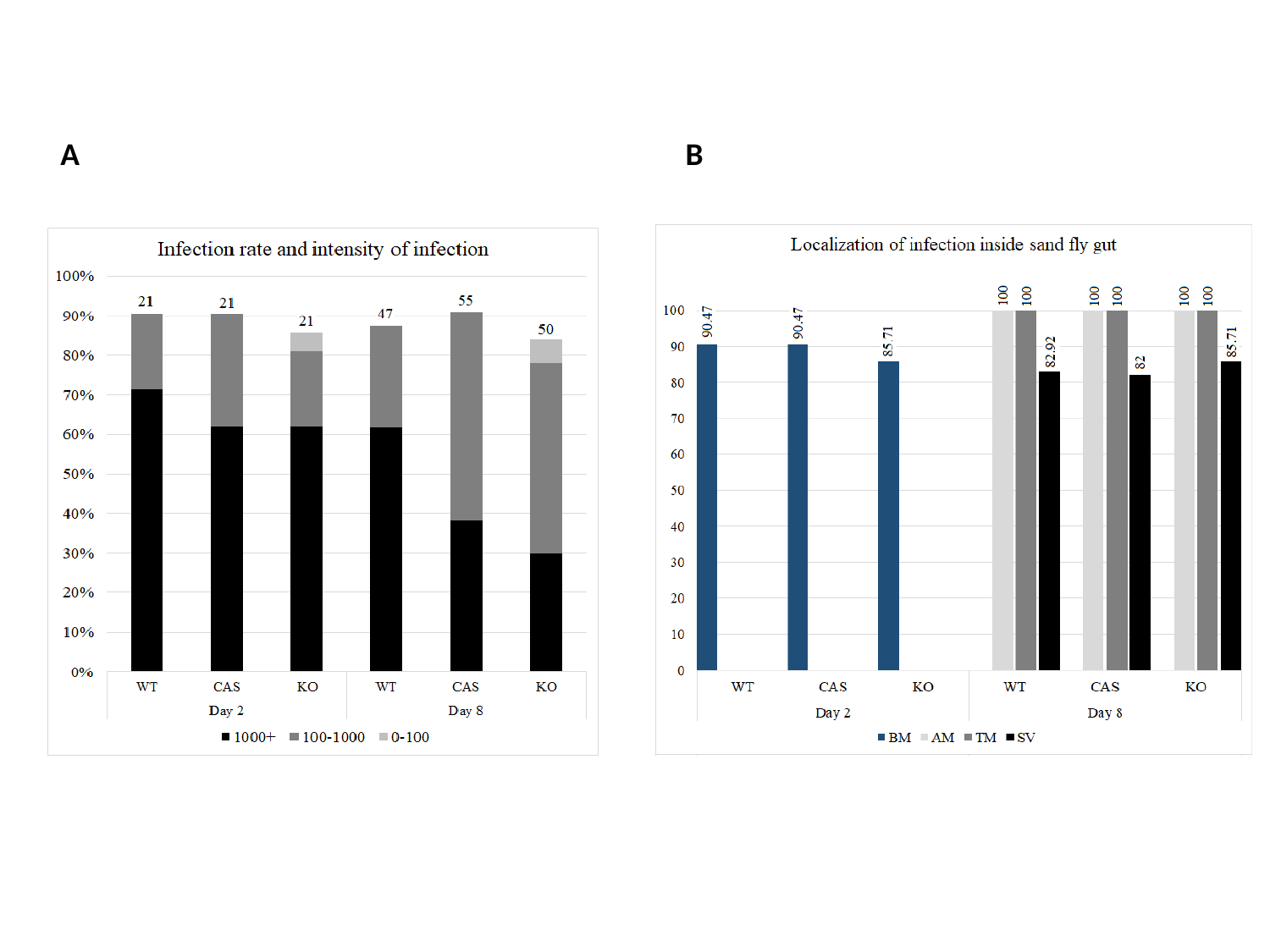

A
B

Supplement: Supplementary file 1 [file pathogens-08-00241-s001.zip › Suppl Fig 3.pptx]
